# Complete plastid genome sequences of *Abeliophyllum distichum* Nakai (Oleaceae), a Korea endemic genus

**DOI:** 10.1080/23802359.2016.1202741

**Published:** 2016-08-31

**Authors:** Hoe-Won Kim, Hae-Lim Lee, Dong-Keun Lee, Ki-Joong Kim

**Affiliations:** a Division of Life Sciences, Korea University, Seoul, Korea;; b Division of Forest Biodiversity and Herbarium, Korea National Arboretum, Pocheon, Korea

**Keywords:** *Abeliophyllum distichum*, endemic genus, plastid genome

## Abstract

The complete plastid genome (plastome) sequences of *Abeliophyllum distichum* Nakai, a monotypic endemic genus of Korea, were determined in this study. The plastome of *A. distichum* was 1,559,825 bp in length (NCBI acc. no. KT274029) and contained a pair of inverted repeat regions (IRa and IRb) of 25,703 bp, which was divided into large single copy region (LSC) region of 86,742 bp and small single copy (SSC) region of 17,834 bp. The overall GC contents of the plastome were 37.8%, and in the LSC, SSC and IR regions were 35.8%, 32.0% and 43.2%. The plastome comprised 129 genes (112 unique), including 78 protein-coding genes, 30 tRNA genes, 4 rRNA genes. Phylogenetic analysis based on 83 genes from 41 plastomes showed that *A. distichum* was most closely related to *Jasminum nudiflorum* with strong support values.

*Abeliophyllum distichum* Nakai is a monotypic endemic genus in Korea (Nakai 1919). It is a deciduous shrub that mainly distributed in the central region of Korea and belongs to the family Oleaceae. *Abeliophyllum distichum* is commonly called white Forsythia, being used as an ornamental plant in Europe and America due to its horticultural value. Phylogenetic relationship among *Abeliophyllum*, *Forsythia* and *Fontanesia* have been controversial in studies based on morphological and cytological characters and chemotaxonomic data (Taylor 1945; Johnson 1957; Harborne & Green 1980; Song & Hong 2013). However, phylogenetic studies of molecular data have demonstrated that *A. distichum* is sister to *Forsythia* and have supported its Korea endemic genus status (Kim et al. 2000; Lee et al. 2007; Kim & Kim 2011). In the present study, we determined the complete plastome of *A. distichum* and it will provide better information regarding the plastome evolution and phylogenetic relationship of Oleaceae.

Plant materials were collected from the natural habitat of Jincheon, Korea. The Genomic DNA was extracted from the fresh leaves using the CTAB method (Doyle & Doyle 1987). The DNA was purified using the ultracentrifugation in cesium chloride/ethidium bromide gradient (Sambrook et al. 1989). Long-range PCR and continuous sequencing of the short fragment using primer walking strategies were utilized for the whole genome amplification. The amplification primers were designed from the plastome sequences of *Nicotiana*, *Panax* and *Jasminum*. Each fragment was sequenced using a series of DNA primer that covers both directions at intervals of 500–800 bp. The PCR products were purified using the MEGAquick-spin kit (iNtRON, Seoul, Korea) and the cleaned products sequenced using an ABI 3730XL automatic sequencer. Sequence fragments were assembled using Sequencer 4.7 (Gene Code Corporation, Ann Arbor, MI). Annotation was performed using the DOGMA (http://dogma.ccbb.utexas.edu/) and BLAST searches. The DNA sample and voucher specimen were deposited in Plant DNA Bank of Korea (PDBK2007-0712) and Korea University Herbarium (KUS 2007-0712), respectively.

The organization, size and gene content of *A. distichum* plastome was similar to most angiosperm plastome (Kim & Lee 2004; Kim et al. 2009). The plastome of *A. distichum* was 155,982 bp in length, including a large single copy (LSC) region of 86,742 bp and a small single copy (SSC) region of 17,834 bp separated by two IR regions of 25,703 bp, respectively (NCBI acc. no. KT274029). The plastome contained 112 unique genes, including 78 protein-coding genes, 30 tRNA genes and four rRNA genes. The six protein-coding genes, seven tRNA genes and four rRNA genes were duplicated in IR region. Sixteen genes contained one intron and two genes (*clp*P and *ycf*3) contained two introns. The overall GC contents of the plastome were 37.8%, and the IR regions (43.2%) were higher in GC content than the LSC and SSC regions (35.8% and 32.0%). Phylogenetic analyses were performed on a data set that included 79 protein coding genes and 4 rRNA genes (aligned length: 83,940 bp) for 41 plastome using RAxML version 8.2.3 and MrBayes version 3.2.5 to infer the phylogenetic position of *A. distichum* (Figure 1). The tree showed that *A. distichum* was most closely related to *Jasminum nudiflorum* among six plastomes in Oleaceae. Their clade was supported strong bootstrap value and posterior probability as 98% and 1.0.

**Figure 1.**
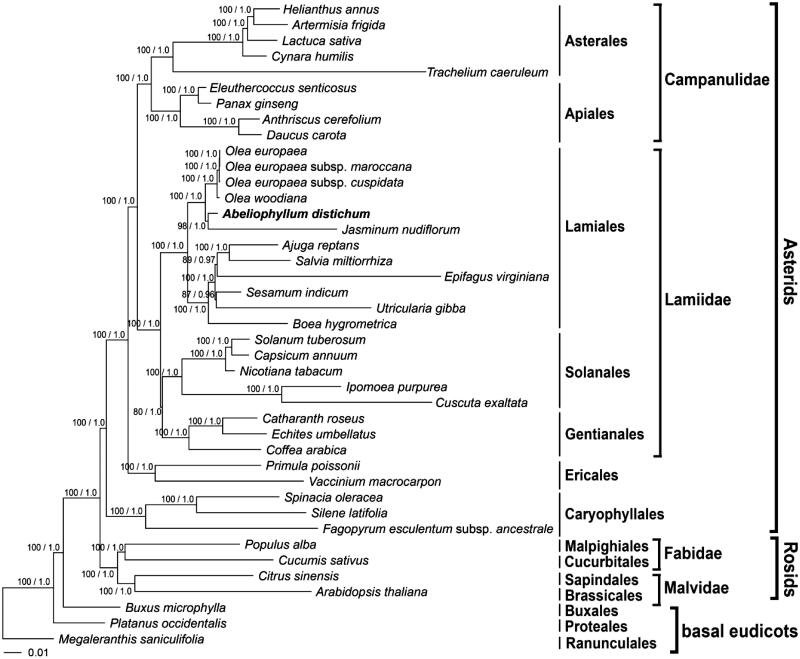
Maximum likelihood (ML) tree based on 83 protein-coding and rRNA genes from 41 chloroplast genomes as determined by RAxML(−ln *L =* −640110.669490). The numbers at each node indicate the ML bootstrap values and Bayesian support values. Scale bar indicates the increment of 0.01 substitution/site. Genbank accession numbers of taxa are shown below, *Abeliophyllum distichum* (KT274029; in this study), *Ajuga reptans* (NC_023102), *Anthriscus cerefolium* (NC_015113), *Arabidopsis thaliana* (NC_000932), *Artemisia frigida* (NC_020607), *Boea hygrometrica* (NC_016468), *Buxus microphylla* (NC_009599), *Capsicum annuum* (NC_018552), *Catharanthus roseus* (NC_021423), *Citrus sinensis* (NC_008334), *Coffea arabica* (NC_008535), *Cucumis sativus* (NC_007144), *Cuscuta exaltata* (NC_009963), *Cynara humilis* (NC_027113), *Daucus carota* (NC_008325), *Echites umbellatus* (NC_025655), *Eleutherococcus senticosus* (NC_016430), *Epifagus virginiana* (NC_001568), *Fagopyrum esculentum* subsp. *Ancestrale* (NC_010776), *Helianthus annuus* (NC_007977), *Ipomoea purpurea* (NC_009808), *Jasminum nudiflorum* (NC_008407), *Lactuca sativa* (NC_007578), *Megaleranthis saniculifolia* (NC_012615), *Nicotiana tabacum* (NC_001879), *Olea europaea* (NC_015401), *O. europaea* subsp. *cuspidata* (NC_015604), *O. europaea* subsp. *Maroccana* (NC_015623), *O. woodiana* (NC_015608), *Panax ginseng* (NC_006290), *Platanus occidentalis* (NC_008335), *Populus alba* (NC_008235), *Primula poissonii* (NC_024543), *Salvia miltiorrhiza* (NC_020431), *Sesamum indicum* (NC_016433), *Silene latifolia* (NC_016730), *Solanum tuberosum* (NC_008096), *Spinacia oleracea* (NC_002202), *Trachelium caeruleum* (NC_010442), *Utricularia gibba* (NC_021449) and *Vaccinium macrocarpon* (NC_019616).
